# Insights From the Supreme Court Decisions: Undesirable Consequences After Minimally Invasive Cosmetic Interventions in Türkiye

**DOI:** 10.1111/jocd.16588

**Published:** 2024-09-20

**Authors:** Mahmut Şerif Yıldırım, Sema Koç Yıldırım

**Affiliations:** ^1^ Department of Forensic Medicine Uşak University Faculty of Medicine Uşak Turkey; ^2^ Department of Dermatology and Venereology Uşak University Faculty of Medicine Uşak Turkey

**Keywords:** complications, cosmetic dermatology, cosmetic legislations, laser, laser hair removal, litigation, medical malpractice

## Abstract

**Background:**

With the increasing demand for cosmetic procedures in recent years, the implementation of some of these procedures by unauthorized persons has led to undesirable results and subsequently to the creation of a large number of case files.

**Aims:**

In this study, it is aimed to retrospectively evaluate the decision texts of the Turkish Supreme Court regarding minimally invasive cosmetic dermatology procedures and to evaluate the reasons and results of the applications reaching the high court in these procedures.

**Methods:**

The Supreme Court's decisions in cases filed due to undesirable consequences caused by minimally invasive cosmetic interventions were scanned using the Supreme Court of Appeals' online database from 2013 to 2023.

**Results:**

The majority of the procedures addressed by the lawsuits are carried out in beauty salons; laser epilation is the procedure that is conducted most frequently, and burns are the most prevalent complication (87.8%, 85.7%, and 77.6%, respectively). As an adverse event, 94.7% (*n* = 36) of burns occurred in beauty centers. Thirteen (26.5%) of the cases in our analysis were carried out by an unauthorized person. When laser epilation and other procedures are considered as two separate categories, in applications due to adverse events of laser epilation, 28 (66.7%) cases were concluded in favor of the defendant.

**Conclusions:**

Complications, especially burns, that occur after laser epilation performed by unauthorized persons in beauty salons constitute a serious caseload, and there seems to be a need for better control mechanisms to reduce this burden.

## 1 Introduction

The frequency of aesthetic and cosmetic dermatological procedures and the demand for these procedures have increased in recent decades, outpacing the number of dermatologists [1, 2, 3, 4]. A significant number of people visit Türkiye, particularly from European and Middle Eastern nations, for aesthetic and cosmetic dermatological operations and interventions as a result of the growing demand for these procedures in the globalizing world [5, 6]. It would be reasonable to assume that as procedure numbers increase, application flaws in this area would become more apparent and that more lawsuits pertaining to this matter will be filed.

Complications and medical malpractices that occur after aesthetic and cosmetic dermatological procedures can cause financial burdens on a wide range of people, including the perpetrator and the victim [7, 8]. In addition to this financial burden, the exhausting effect of litigation processes should also be taken into account. The psychological effects on healthcare professionals and patients on both sides of the case processes may also become an issue in the future when the protracted litigation processes and objections to the cases. Also, the pressure of concluding these objections may be added to the wear and tear experienced in each case process.

In Türkiye, civil and criminal courts give decisions separately from each other, within a judicial process similar to continental Europe. Objections to decisions made by first instance courts serving certain regions in both criminal and civil trials are decided at a higher level in the Regional Courts of Justice established within the framework of compliance with the European Union [9]. At the last step in the ordinary legal path is the Court of Cassation of the Republic of Türkiye, as the Supreme Court whose decisions are binding on all other courts [10].

Since the Supreme Court's rulings set precedent, they can serve as a guide for judges, attorneys, and prosecutors during trials as well as for medical professionals, patients, and other healthcare providers in non‐trial situations. In this study, it is aimed to evaluate the decision texts given by the Court of Cassation of Türkiye on minimally invasive cosmetic dermatology procedures retrospectively and to evaluate the reasons and results of the applications that reached the high court in these procedures.

## 2 Materials and Methods

In this study, the decisions given by the Court of Cassation of Republic of Türkiye were investigated retrospectively. The Supreme Court's decisions in cases filed due to undesirable consequences following minimally invasive cosmetic interventions were scanned using the Supreme Court of Appeals' online database [11] throughout a decade period from 2013 to 2023. During the search, the keywords “*beauty* AND *center*; OR *beauty* AND *salon*; OR *cosmetology* OR *dermo‐cosmetics* OR *botox* OR *filler* AND *injection*; OR *dermatology* OR *laser* AND *epilation* OR *peeling* OR *cosmetic* AND *intervention*; OR *hair* AND *transplantation*; OR *aesthetic* AND *intervention*” were used, and a total of 311 decisions were obtained. Out of these 311 decisions, a total of 49 cases were included in the study after excluding cases where the subject of the case did not include cosmetic procedures, the subject of the case was different, repeated decision texts, dental procedures, and major surgical procedures.

The year of admission of the case to the Supreme Court, the year of the decision by the Supreme Court, the cosmetic dermatological procedure in question, the information about the defendant, the decision of the first instance court, the decision of the Supreme Court, the chamber to which the court that issued the decision belongs, and the reasons for reversal in cases where the Supreme Court has decided to reverse the decision of the first instance court were gathered and recorded from the decision texts issued by the Supreme Court. In addition to frequency and percentage calculations from the obtained data, Fisher's exact test was used to determine whether there was a statistically significant difference between the complications that developed, and the decision given according to the place where the procedure was performed, and the procedures performed. All statistical analyses were performed using SPSS 22.0.

No ethical board decision required for the study, since data used in the study is derived from an open database.

## 3 Results

**FIGURE 1:**
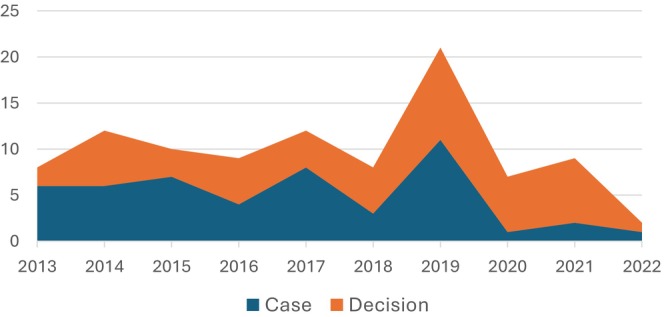
Numbers of case admissions and decisions of the Supreme Court by years.

The highest number of the Supreme Court appeals and decisions were in 2019 and the lowest in 2022 (Figure 1).

It was noted that the majority of the procedures addressed by the lawsuits are carried out in beauty salons; laser epilation is the procedure that is conducted most frequently, and burns are the most prevalent complication (Table 1).

**TABLE 1:** Information about procedures, adverse events, and defendants of the cases.

		*n*	%
Procedure	Laser epilation	42	85.7
	Hair transplant	1	2.0
	Chemical peeling	1	2.0
	Injection	1	2.0
	Tattoo removal	1	2.0
	Solarium	1	2.0
	Lipolysis	1	2.0
	Waxing	1	2.0
Adverse event	Burn	38	77.6
	Hypertrichosis	2	4.1
	Unsatisfying results	2	4.1
	Pigmentation disorders	7	14.3
Defendant	Beauty/cosmetology center	43	87.8
	Hospital	4	8.2
	Physician's private clinic	2	4.1
	Total	49	100.0

When the adverse events were divided into beauty centers and other health institutions according to where they occurred, a statistically significant difference was found in terms of the frequency of complications (*p* < 0.001). As an adverse event, 94.7% (*n* = 36) of burns occurred in beauty centers, while only two occurred in other institutions. While applications to the Supreme Court with complaints of hypertrichosis were made only from beauty centers, unsatisfying results were only the subject of complaints after procedures performed in other health institutions. Two (28.5%) of the pigmentation problems happened in other healthcare facilities, whereas five (71.4%) happened in beauty centers.

While three‐quarters of the decisions brought to the Supreme Court were penal cases and one‐quarter were civil cases, 61.2% (*n* = 30) of the first instance court decisions were in favor of the defendant (Table 2).

**TABLE 2:** Information about legal cases and decisions.

		*n*	%
Legal chamber	Civil	12	24.5
	Penal	37	75.5
First decision	In favor of the defendant	30	61.2
	In favor of the complainant	19	38.8
Supreme court decision	Approval	17	34.7
	Reversal	32	65.3
Causes of reversal	Court without jurisdiction	1	2.0
	Insufficient expert witness process/report	27	55.1
	Judgment contrary to expert opinion	1	2.0
	Low compensation	2	4.1
	High compensation	1	2.0
Insufficiencies of expert witness process	Expert with little/no experience in the field	3	6.1
	Insufficient evaluation	18	36.7
	No expert witness	6	12.2
Unauthorized personnel use of the defendant	Yes	13	26.5
	No	36	73.5
	Total	49	100.0

In 13 of the case files (26.5%), it was noted that the person performing the procedure did not have the necessary training and competence to perform the procedure in question. Twelve (92.3%) of the individuals who carried out unauthorized treatments were employed by beauty salons, while one (8.7%) was employed by other medical facilities. It was observed that 11 of these unauthorized procedures (84.6%) were laser interventions performed by a non‐physician who did not have training and competence in using the laser.

When laser epilation and other procedures are considered as two separate categories, in applications due to adverse events of laser epilation, 28 (66.7%) cases were concluded in favor of the defendant and 14 (33.3%) cases were concluded in favor of the complainant, while in other procedures these numbers were 2 (28.6%) and 5 (71.4%), respectively.

In 26 of the 27 cases where the Supreme Court used the inadequacy of the expert report as the basis for reversal of the decision, the procedure in question was laser epilation. In the other procedures, it was seen that in one case, a reversal decision was made due to a decision contrary to the expert report. It was determined that the reasons for reversal of the decision showed statistically significant differences according to the procedures performed (*p* = 0.023).

## 4 Discussion

Examining the procedures covered by the study that are the subject of the lawsuit, we see that the great majority of them involve complaints following laser epilation. When all minimally invasive cosmetic procedures are evaluated, similar to our study, studies in the literature show that laser epilation is the most frequently litigated procedure, and burns are suggested as the most common adverse event [4].

In a study examining the financial impacts of the Supreme Court decisions, including major surgeries and dental practices, over a 5‐year period, it was stated that beauty centers were included in the Court of Cassation files at a rate of 22% [7]. Since major surgeries cannot be performed outside of a hospital setting, the rate of hospitals in this study was thought to be higher than in our study. Nonetheless, similar to our assessment, it is seen that laser epilation cases rank highest among the interventions under consideration in Yalçın‐Balçık and Çakmak's study, even when other procedures are taken into account. The same study shows that burns are the second most common injury after deformities [7].

Burns were found to be the most often reported adverse event by the complainants in our study. In a study evaluating only cosmetic laser applications through forensic medicine files, burns were reported as the most common adverse event [12]. In the study by Kar et al. [13], in which they evaluated the cases applied to the Forensic Medicine polyclinic, it was stated that the most common finding was pigmentation disorders. In contrast to our study, which found scars and post‐burn pigmentation disorders rather than the burn itself, the majority of the cases in the study were assessed months to 2 years after the occurrence, and pigmentation disorders were assumed to be the most common finding in their study. Similarly, pigmentation disorders due to burns have been reported as the most common adverse event after laser applications [14]. In a US‐based study where medical malpractice cases of laser procedures were evaluated retrospectively, the total pigmentation disorders were more frequent than burns, similar to the study by Kar et al. [8, 13].

In our country, medical malpractice cases are tried in criminal courts as the crime of wounding by negligence. As a separate process, civil courts also conduct trials for compensation claims of individuals. Depending on the verdict in the criminal case, sometimes the parties can agree on compensation among themselves or through mediators and there may not be an application to a civil court. In our study, the fact that the number of cases decided in penal chambers is higher than that seen in civil chambers seems to be consistent with this information. In the study by Yalçın‐Balçık and Çakmak, which included all aesthetic interventions, including major surgeries, it was seen that four‐fifths of the cases were made up of civil chamber decisions [7]. It is thought that this major difference is due to the fact that we did not include major surgical procedures as a basis in our study, unlike them, and the difference in the time interval of the files included in the study.

More than half of the Supreme Court's reversal grounds are expert witness process insufficiencies. A minimum of 5 years of current experience in the appropriate profession and expert witnessing training are requirements for those who wish to testify as experts in courts of Türkiye [15, 16]. Forensic medicine specialists, as official experts, often prepare expert witness reports together with dermatologists who are experts in the field. Experts expressing opinions outside their fields, acting as expert witnesses despite not having completed sufficient experience, or expressing opinions without providing sufficient scientific justification in their reports/statements may result in the high court taking a reversal decision due to the ineffectiveness of the expert witness process.

In our country, according to the Regulation on Amendments to the Regulation on Business Opening and Working Licenses [17], the devices that are permitted to using by beauticians in beauty salons for hair removal applications are intense pulsed light (IPL) with 600–1200 nm wave range and diode laser that is produced only for epilation with the maximum energy limit of 20 j/cm^2^. Nevertheless, thirteen (26.5%) of the cases in our analysis were carried out by an unauthorized person. Although it is not detailed in the file contents, the definition of an unauthorized person could be in three ways: a beautician who does not use an appropriate device (i), a person who uses an appropriate device but is not a beautician (ii), a person who is not a beautician and uses an inappropriate device (iii). In another study from Türkiye including 14 cases where forensic medical evaluation was performed to evaluate the injuries caused by laser epilation, it was determined that 12 of the cases (85.7%) were performed in beauty centers by unauthorized operators, and only two cases were performed by authorized physician extenders under physician supervision [13]. This study was conducted in a confined area and includes examinations conducted during the first instance trials, which may be the reason why a higher proportion of people utilized lasers despite not being permitted than in our study.

In the current legislation, some procedures are accepted as procedures to be performed only by a physician, some procedures can be performed by a physician or by auxiliary health personnel under the supervision of a physician, and some procedures can be performed by non‐physician personnel who have received special training. Cases where the physician is held responsible with vicarious liability for procedures performed by non‐physician personnel, taking into account the nature of his/her supervision, occupy a large place in medical malpractice files in our country as well as all over the world [18, 19, 20]. Even if non‐physician personnel have the necessary training for the application, they are not successful in managing the complications that may arise during the application [21]. The fact that all of these centers were private enterprises and that over one‐quarter of the decisions highlighted operators who were either unsupervised by physicians or lacked the necessary training to demonstrate their competence in the procedure can be explained by the fact that more and more centers are opening every day to capture a share of the expanding market. These centers are also attempting to offer services beyond their capacity out of financial greed, and unfortunately, control mechanisms are insufficient. Considering that there is a greater risk of adverse events and lawsuits in procedures performed by non‐physicians, even under appropriate supervision, it would not be wrong to predict that personal, psychological, and financial damages would increase even more under these conditions where the physician is not involved in the picture at all [14, 19, 21]. In a retrospective study conducted on forensic medicine files, it was stated that in 6 out of 10 cases, the operator did not have the necessary competence in the examinations performed after cosmetic laser applications, and that lack of supervision stood out as a problem [12].

Sacchidanand and Bhat divided patients who underwent cosmetic procedures into five groups according to the reasons for needing the procedure and classified one of these groups as those who needed the procedure due to psychiatric problems [22]. It is necessary to review the psychiatric evaluations and past cosmetic procedures of people in this group before the procedure. However, these examinations are not performed in centers that are not under physician supervision or that cannot access patient files because they are not part of the national health network. Given that our nation serves as a major medical tourism hub, it becomes unfeasible to obtain medical history information for visitors from other nations. In addition to making more patients dissatisfied with the procedure overall, this condition pushes patients with psychiatric disorders to engage in potentially exacerbating procedures rather than receiving the proper treatment. Naturally, a portion of these patients also bring legal action in court, which implies that there will be more cases in the future. Since the Supreme Court rulings concealed the identities of the parties involved, we were unable to evaluate this matter. However, we believe that this issue is one of the most fundamental topics that needs to be investigated.

There are several limitations in our study. The first is that the number and diversity of files are relatively small. Another limitation is the insufficient explanations in the content of files, for instance, unauthorized persons or laser devices. Also, insufficient information about the complainant and the defendant is not available due to confidentiality of personal information.

In conclusion, according to the information obtained from this study, although certificated beauticians are authorized to perform laser epilation (IPL and diode laser) in beauty salons in our country, applications performed by incompetent people in these workplaces cause undesirable consequences, especially burns. Moreover, considering that unauthorized transactions other than laser epilation are carried out in these workplaces, these situations suggest the inadequacy of the control mechanism. Strengthening the control mechanism may be a suggestion, and not allowing any operations, including laser epilation, to be performed without a physician's supervision in these workplaces may also be one of the solutions.

## Ethics Statement

No ethical board decision required for the study, since data used in the study is derived from an open database.

## Conflicts of Interest

The authors declare no conflicts of interest.

## Data Availability

Data derived from public domain resources.
